# B-Lines in the Assessment of Interstitial Lung Disease Associated with Scleroderma: The Role of Handheld Devices

**DOI:** 10.3390/diagnostics14212397

**Published:** 2024-10-28

**Authors:** Codrina Ancuța, Cristina Pomirleanu, Ecaterina Gănceanu, Milena Adina Man, Eugen Ancuta, Paraschiva Postolache

**Affiliations:** 1 Department of Rheumatology, School of Medicine, Grigore T. Popa University of Medicine and Pharmacy, 700115 Iasi, Romania or codrina_ancuta@yahoo.com (C.A.); or crismun@yahoo.com (C.P.); 2 Rheumatology 2 Department, Rehabilitation Clinical Hospital, 700664 Iasi, Romania; 3 Department of Pulmonology, Iuliu Hatieganu University of Medicine and Pharmacy, 400347 Cluj Napoca, Romania; manmilena50@yahoo.com; 4 Research Department, Elena Doamna Clinical Hospital, 700398 Iasi, Romania; 5 Internal Medicine Department, Grigore T. Popa University of Medicine and Pharmacy, 700115 Iasi, Romania; par.postolache@umfiasi.ro; 6 Respiratory Rehabilitation Clinic, Rehabilitation Clinical Hospital, 700664 Iasi, Romania

**Keywords:** systemic sclerosis, lung ultrasound, handheld probe, B-lines, high-resolution CT (HRCT)

## Abstract

**Background**: Timely detection and aggressive management of interstitial lung disease (ILD) in systemic sclerosis (SSc) are essential to improving outcomes and reducing risks of irreversible lung injury. **Objective**: to explore the usefulness of an ultraportable ultrasound device for the management of SSc-related ILD and to compare it with clinical and instrumental data. **Methods**: A total of 19 consecutive SSc patients underwent a comprehensive pulmonary evaluation: clinical, pulmonary function tests (PFTs) (spirometry, DLCO), lung CT (1.5 mm slice thickness reconstruction; HRCT), and lung ultrasound (LUS). A total score was calculated based on the number of color-coded B-lines recorded for each lung sliding. B-lines were analyzed against dyspnea, cough, Velcro, CT imaging (Warrick’s score), and PFTs. Global and subgroup analysis were performed (diffuse versus limited cutaneous SSc, Warrick’s < 7 versus >7). **Results**: Symptomatic lung involvement with varying degrees of dyspnea was reported in about 74% of cases (functional NYHA > 2 in more than half), chronic dry cough in one-third, Velcro rales in 42%. A total of 84.24% were classified as SSc with ILD on CT imaging. Statistically significant mild-to-moderate correlations between B-lines and clinical manifestations were demonstrated, as well as PFTs and Warrick’s scores (more B-lines, lower pulmonary function, but higher extent and severity on CT) (*p* < 0.05); there were differences between SSc patients without and with ILD in terms of the number and distribution of B-lines (*p* < 0.05), as well as different B-lines patterns and numbers in diffuse versus limited SSc (*p* < 0.05). **Conclusions**: Ultraportable handheld LUS is a promising method suitable for the management (screening, early detection, and evaluation) of SSc patients.

## 1. Introduction

Interstitial lung disease (ILD) is a common complication of systemic sclerosis (SSc), a rare autoimmune disease characterized by excessive collagen deposition in various tissues and organs. This process leads to progressive fibrosis and vascular damage, which are hallmark features of the disease. ILD significantly contributes to morbidity and mortality in SSc patients [1]. The exact mechanisms underlying the development of ILD in SSc are not fully understood but likely involve a combination of immune dysregulation, endothelial dysfunction, abnormal fibroblast activity, and oxidative stress [1,2,3,4]. ILD in SSc can significantly affect the prognosis and quality of life of affected individuals, often contributing to increased morbidity and mortality [5,6,7,8].

The clinical manifestations of SSc-related interstitial lung disease can vary widely, from asymptomatic cases detected incidentally on imaging studies to severe respiratory failure requiring mechanical ventilation. This variability underscores the importance of early detection and monitoring to manage disease progression effectively [5,6,7,8]. Common symptoms include a chronic dry, nonproductive cough, shortness of breath, oxygen desaturation with ambulation (a drop of over 3% in pulse oximetry), and, in advanced stages, hypoxemia at rest. Additionally, Velcro-like crackles can often be heard on pulmonary auscultation [1,5,6,7,8]; The diagnosis of SSc-ILD typically follows a standard algorithm that includes clinical evaluation, pulmonary function tests (PFTs), high-resolution computed tomography (HRCT) of the chest, and, in some cases, lung biopsy to further characterize the underlying histopathology [5,6,7,8].

Timely detection and aggressive management of interstitial lung disease (ILD) in systemic sclerosis (SSc) and other systemic immune-mediated disorders are essential for improving outcomes and minimizing the risk of irreversible lung damage [3,4,5,6,7,8]. Early intervention, particularly in scleroderma-induced ILD, can significantly improve prognosis by slowing disease progression, managing symptoms, and enhancing quality of life. Available therapies include immunosuppressive agents such as mycophenolate mofetil and cyclophosphamide, antifibrotic drugs like nintedanib, and supportive treatments including oxygen therapy and pulmonary rehabilitation.

Without early detection and treatment, ILD can result in irreversible lung damage. Initiating therapy promptly can help reduce inflammation and fibrosis, preventing or delaying severe respiratory impairment. Regular monitoring using pulmonary function tests and imaging is critical for tracking disease progression and adjusting treatment plans accordingly.

Periodic assessment, including identification of patients at risk for lung fibrosis, as well as the use of validated pulmonary function tests and imaging, is vital for evaluating treatment response. Collaboration between primary care, pulmonology, radiology, and rheumatology, ideally within a tertiary ILD center, ensures comprehensive patient care and optimized outcomes [5,6,7,8,9]. Pulmonary function tests are often used to evaluate ventilatory and diffusion dysfunction in patients with ILD, based on spirometry, lung volume measurements, and single-breath carbon monoxide diffusion capacity (DLCO) [5,6,7,8,9]. Validated parameters including forced expiratory volume (FEV), forced vital capacity (FVC), residual volume (RV), as well as DLCO are consistently important to demonstrate the restrictive respiratory pattern that is suggestive for fibrotic involvement, while the changes over time of these tests usually reflect the progressive fibrotic pattern of ILD related to SSc [5,6,7,8,9].

Lung HRCT remains the gold standard for the management of SSc-related ILD, as it is sensitive enough to detect subclinical stages of ILD, is validated to evaluate the extent and severity of parenchymal lung involvement (using standardized methods and scores such as Warrick’s score [10]), to identify the progression of pulmonary fibrosis and patients with progressive phenotype, and to monitor treatment efficacy [1,2,3,4,5,6,7,8,9]. Interstitial changes or markings, reticulations or reticular changes, traction bronchiectasis, architectural distortion, and honeycombing remain key lesions used to highlight interstitial lung disease [3,4,5,6,7,8,9,10,11].

Quite recently proposed as a new imaging technique, lung ultrasound (LUS) is suitable for the screening, exceedingly early detection (preclinical stages), diagnosis, and monitoring of pulmonary fibrosis associated with connective tissue disorders including SSc [11,12,13,14,15]. Although there is no consensus regarding the role of LUS in the diagnosis and/or prognosis of SSc-ILD, several publications have already reported a substantial correlation between LUS and HRCT findings, PFTs, and even DLCO values, as well as severity scores and predictive value [12,13,14,15,16,17,18].

B-lines and pleural line abnormalities are two main findings used to characterize ILD on LUS using both convex and sector arc probes with frequencies ranging from 3.5 to 10 MHz [19,20,21]. According to the recent OMERACT definition, B-lines account for discrete laser-like vertical hyperechoic reverberation artefacts that arise from the pleural line surface to the edge of field [9,19,20]; they move synchronously with lung sliding and are caused by the reflection of ultrasound waves by interlobar septal thickening from fluid or fibrosis [9]. B-lines are widely used for screening systemic sclerosis-related interstitial lung disease due to their high sensitivity and negative predictive value. The total number of B-lines is significantly higher in patients with interstitial lung involvement compared to those without. Additionally, B-lines can detect both new-onset and worsening pulmonary fibrosis, making them a valuable tool for monitoring disease progression [9,10,11,12,13,14,15,16,17,18,19,20]. Early pulmonary fibrosis is characterized by the presence of only a few B-lines, while advanced stages are marked by more confluent B-lines [9,10,11,12,13,14,15,16,17,18,19,20]. Although no standardized scanning protocol exists for SSc-ILD, limited scanning of the postero-basal chest (between the scapula and diaphragm) appears to provide the most sensitive and rapid assessment of interstitial lung disease, offering an optimal balance of image quality and efficiency [9,13,15]. When evaluating systemic sclerosis (SSc) and ultrasound findings such as B-lines, it is important to consider various differential diagnoses. For SSc, differential diagnoses include mixed connective tissue disease (MCTD), rheumatoid arthritis (RA) with interstitial lung disease, dermatomyositis/polymyositis, sarcoidosis, and idiopathic pulmonary fibrosis (IPF). In the context of ultrasound findings, B-lines may indicate conditions beyond SSc-ILD, such as heart failure with pulmonary edema, pneumonia, acute respiratory distress syndrome (ARDS), pulmonary fibrosis, and pleural effusion. These differential diagnoses should be carefully considered to ensure accurate assessment and management of lung involvement in patients with SSc and other related conditions [9,13,15,20].

Lung ultrasound offers several clear advantages over more sophisticated imaging techniques such as HRCT, MRI, and PET-CT. These benefits include being non-invasive, portable, radiation-free, and low-cost, making LUS a practical alternative for lung assessment in various clinical settings [9,22,23].

Handheld lung ultrasound (HH-LUS) devices are compact, portable tools specifically designed for the rapid assessment of lung conditions at the point of care, enabling clinicians to perform bedside evaluations with ease and efficiency [22]. These handheld devices facilitate quick, dynamic visualization of lung anatomy and functions like lung sliding. Their growing use in diverse clinical settings is attributed to several advantages, including portability, ease of operation, real-time imaging, cost-effectiveness, and the significant benefit of minimizing radiation exposure compared to conventional imaging methods [9,22,23]. HH-LUS devices have demonstrated comparable accuracy to cart-based machines for lung and pleural assessments, as previously shown in studies. This makes them a reliable alternative for point-of-care evaluations without sacrificing diagnostic precision [23]. Moreover, a recent study aimed to evaluate features such as ease of use, image quality, and overall expert satisfaction with available HH-LUS devices. The findings provided insights into which device is best suited for routine clinical practice, highlighting the importance of balancing functionality with user experience in selecting the most appropriate tool for lung assessment [23].

The aim of the current study was to evaluate the usefulness of an ultraportable ultrasound device (Lumify™ 12-4 Android/Linear Array Transducer, Philips Healthcare, Romania, Bucharest) in the assessment of systemic sclerosis-related interstitial lung disease and to compare its findings with standard clinical and instrumental data.

## 2. Materials and Methods

### 2.1. Study Population

We conducted a retrospective observational study on 19 consecutive patients with systemic sclerosis who met the 2013 ACR/EULAR classification criteria. These patients attended a tertiary university rheumatology center at the Clinical Rehabilitation Hospital in Iasi, Romania (EUSTAR Center 162).

All patients with systemic sclerosis (SSc) hospitalized between 1–31 March 2023 were eligible for the study, regardless of their disease subset (diffuse or limited cutaneous SSc), respiratory impairment (symptomatic or asymptomatic), prior lung assessment, or treatment. However, patients with lung or heart diseases unrelated to SSc were excluded. Additional exclusions included those with lung cancer, interstitial fluid, asthma, chronic obstructive pulmonary disease (COPD), kyphoscoliosis, dyspnea requiring oxygen, and connective tissue diseases other than SSc.

### 2.2. Data Collection

The following data were collected: (i) demographic information; (ii) clinical subset of SSc, classified by the extent of skin involvement into limited or diffuse forms, the modified Rodnan skin score (mRSS) ranging from 0 to 51, history of Raynaud’s phenomenon, digital ulcers, non-productive dry cough, dyspnea, and presence of Velcro rales on pulmonary auscultation; (iii) laboratory data, including autoimmune serology for antinuclear antibodies (ANAs), anti-centromere antibodies (ACA), anti-topoisomerase I (anti-SCL70) antibodies, and complement levels.

The EUSTAR activity score and Medsger Lung Severity Scale (MLSS) were calculated for each study participant. The Medsger severity scale evaluates systemic sclerosis involvement across multiple organs, while the MLSS specifically assesses lung damage severity. Points are assigned based on pulmonary function tests, CT scans, and echocardiography parameters, with the scale ranging from 0 (normal lung function: DLCO > 80%, FVC > 80%, no fibrosis on X-rays, and systolic pulmonary artery pressure [SPAP] > 30 mmHg) to 4 (end-stage lung disease requiring oxygen) as shown in Table 1 [24].

All patients underwent pulmonary function tests and lung ultrasound. Lung CT scans were performed only in patients who had not undergone an assessment within the previous 3 months.

### 2.3. Pulmonary Function Tests

Lung function tests were performed by a single trained assessor using internationally validated protocols and reference values. The primary focus was on forced vital capacity (FVC in mL and %), forced expiratory volume in the first second (FEV1 in mL and %), and the FEV1/FVC ratio (%), all assessed through spirometry using COSMED pulmonary function testing (PFT) equipment. This equipment included capabilities for spirometry, lung volumes, DLCO, and body plethysmography. Standard pulmonary DLCO measurements were also taken and corrected for hemoglobin levels.

### 2.4. Lung CT

CT lung examinations were performed using a standard protocol on a Toshiba Aquilion RXL 16 CT Scanner (DLP range per scan: 76–786 mGy⋅cm, translating to a lung dose of 1–11 mGy and an effective dose of 1–8 mSv, based on volumetric CT dose index-to-organ and effective dose conversion factors for adult chest CT) in the Radiology Department. No iodine contrast agents were used (native CT), and all scans were reconstructed with a slice thickness of 1.5 mm to simulate high-resolution (HRCT) imaging. The images were evaluated following the Fleischner Society glossary, focusing on lesions such as ground-glass opacities, consolidation, septal/subpleural lines, irregular pleura, nodules, subpleural cysts, architectural distortion, traction bronchiectasis, honeycombing, atelectasis, pleural effusion, and mosaic attenuation patterns. Extension, severity, and global scores were assessed using Warrick’s semi-quantitative scoring method, which is validated for scleroderma-related ILD (see Table 2) [10,25]. The total score ranges from 0 to 30, with a score greater than 7 indicating significant interstitial lung involvement. Each HRCT image was independently assessed by an experienced radiologist and a pulmonary disease specialist, who also calculated the Warrick score for each case.

### 2.5. Lung Ultrasound

Lung ultrasound was performed using the Philips Lumify™ L12-4 transducer, (Philips Healthcare, Romania, Bucharest) paired with a portable Samsung Galaxy tablet and the Lumify app [26], by a single observer (G.E., an LUS-trained rheumatologist) who was blinded to clinical data, PFTs, and previous lung scans. The linear transducer was set to a pulmonary configuration, recording 4 s loops of 12 lung regions to quantify B-lines, with each region color-coded based on the number of B-lines detected. A standardized protocol was followed, assessing three regions on both sides of the thorax: anterior (midclavicular line), lateral (midaxillary line), and posterior (paravertebral line), with each divided into upper and lower subregions. The anterior chest was examined with the patient in a lying position, the lateral chest in a supine position, and the posterior chest with the patient seated, facing away from the operator [26].

**Figure 1:**
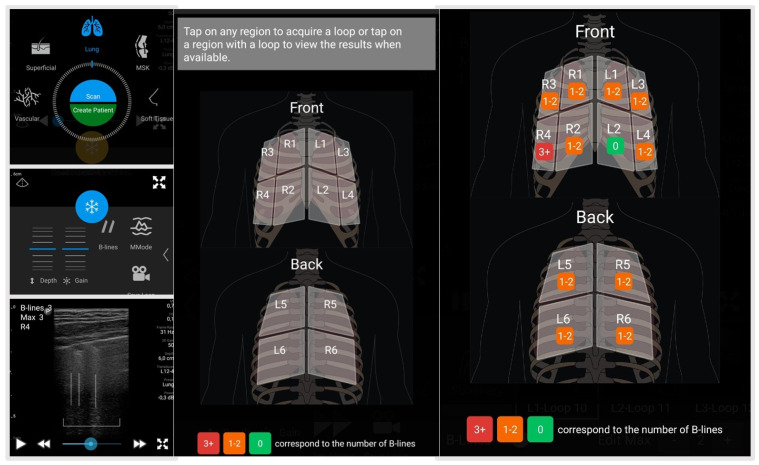
Lung ultrasound scan protocol and mode selection in this study (from [26]).

The distribution and anatomical landmarks are summarized in Table 3, with the typical scanning mode illustrated in Figure 1. B-lines in each thoracic region were counted according to the validated OMERACT definition [9]. A color-coded system was applied: “green 0” indicating no B-lines, “orange 1–2” representing 1 to 2 B-lines, and “red 3+” denoting 3 or more B-lines. A region was considered positive for ILD if there were ≥3 B-lines in a single region or >5 in adjacent spaces. Additionally, the appearance of 10 or more B-lines in a region, known as the “all-white” pattern, was noted [26]. A B-line score ranging from 0 to 24 was calculated by summing 1 point for each region with a red color code across the 12 scanned regions. Regions with green or orange color codes did not receive any points [26].

### 2.6. Statistical Analysis

The data were entered into a Microsoft Excel database, and statistical analysis was conducted using Excel Office 2019 (University of Medicine and Pharmacy “Grigore T. Popa” Iasi, Romania). Means ± standard deviation (SD) and frequencies (%) were calculated. The Spearman Rank Correlation Coefficient was used to assess the relationships between LUS results (B-lines) and clinical variables, PFTs, and Warrick’s CT score. Subgroup analyses were performed comparing diffuse versus limited cutaneous SSc and Warrick’s score < 7 versus >7. A *p*-value of less than 0.05 was considered statistically significant (*p* < 0.05).

All patients provided written informed consent prior to their enrollment in our study.

## 3. Results

### 3.1. Patients Characteristics

The demographic, clinical, and serological data of the patients enrolled in this study are summarized in Table 4. As expected, women were twice as likely to be affected by SSc compared to men, and nearly two-thirds of the patients had the diffuse cutaneous SSc subtype (dcSSc). More than half of the patients had a disease duration of less than 5 years. All patients exhibited peripheral vascular involvement, with the majority presenting with mild Raynaud’s phenomenon (Raynaud condition score ranging from 1 to 5) and mild to moderate skin involvement (mRSS below 20 out of 51 in up to 90% of cases). Despite the classification of Raynaud’s phenomenon as mild, 42% of patients (n = 8) had digital scarring at the time of presentation, and a history of digital ulcers was reported in 21% of cases.

Symptomatic lung involvement has been detected, with varying degrees of dyspnea being reported in up to 74% of cases (NYHA functional > 2 in more than half), chronic dry cough in one-third, and also Velcro rales in 42% patients. Seven patients had normal chest X-rays, while on the CT scan only three of them presented with a normal lung CT scan (SSc without ILD); 16 were classified as SSc with ILD as demonstrated on CT imaging.

Positive ATA were detected in 14 cases, ACA in three cases, two cases being seronegative for ACA and ATA; as a marker of disease activity, low serum complement levels were shown in five cases (26.32%).

Data on the assessment of lung damage are presented in Table 5.

### 3.2. Pulmonary Function Tests

FVC was 92.7 ± 23.65 across the entire patient cohort, with significant differences between patients without interstitial lung disease (103 ± 20) and those with SSc-ILD (85 ± 15) (*p* < 0.05). Additionally, substantial differences were observed between patients with severe SSc-ILD (88 ± 19) and those with limited ILD associated with SSc (97 ± 18) (*p* < 0.05).

Regarding DLCO, a severe decrease was found in 21% (n = 4) of cases, moderate in 21% (n = 4), mild in 10.53% (n = 2), while 47.36% (n = 9) had normal DLCO values. The mean DLCO for the entire cohort was 82 ± 19, with significantly lower values in patients with extensive ILD (55 ± 12) compared to those with limited ILD (78 ± 15) and those without ILD (85 ± 18) (*p* < 0.05).

As expected, patients with SSc-ILD (n = 16) had significantly lower PFT results, including DLCO, compared to those without ILD (*p* < 0.05). A similar trend was observed in patients with diffuse cutaneous SSc (dcSSc) versus limited cutaneous SSc (lcSSc), with significantly lower values in those with dcSSc (*p* < 0.05).

### 3.3. HRCT Assessment

Ground-glass opacifications were the most common findings, observed in 78.95% (n = 15) of cases, followed by honeycombing in 26.23% (n = 5), septal/subpleural lines in 21.05% (n = 4), and subpleural cysts in 10.53% (n = 2). Interstitial lung disease (ILD) was detected in 84.21% (n = 16) of cases, with non-specific interstitial pneumonia (NSIP) identified in 57.89% and usual interstitial pneumonia (UIP) in 31.58%. The average total Warrick score across the group was 5.57 ± 4.47, with the extension score averaging 2.26 ± 1.91 and the severity score 3.31 ± 2.84.

### 3.4. LUS Assessment

**Figure 2:**
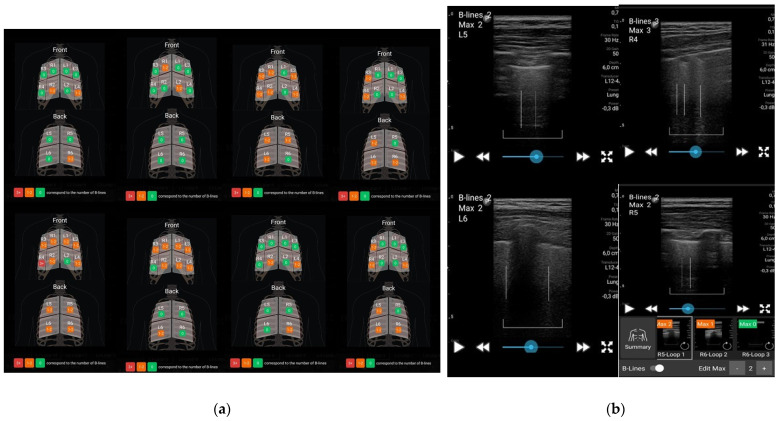
LUS imaging: scanned regions with color code assessment (**a**) and B-lines within lung sliding (**b**).

B-lines were detected in all cases, with an average of 5.42 ± 2.60 out of a maximum of 24 points across 12 regions examined, with the most common locations being the lower segments: R2, L4 (anterior), and R6 (posterior). Examples of ultrasound B-lines reports are illustrated in Figure 2. The majority of scanned regions were coded as “green”, with an average of 6.68 ± 2.61 B-lines. Regions coded as “orange” had an average of 5.21 ± 2.46 B-lines, while regions with three or more B-lines, coded as “red”, had an average of 0.10 ± 0.44.

Subgroup analysis revealed significantly higher B-line scores in patients with SSc-related ILD compared to those without.

### 3.5. LUS in Relation to Clinical, Functional, CT Scan

Significant correlations were observed between the number of LUS B-lines and clinical pulmonary manifestations, including cough (r = 0.2), dyspnea (r = 0.16), and Velcro crackles (r = 0.25). Additionally, correlations were found with fibrosis extension and severity as measured by the Warrick score (r = 0.32), Warrick extension (r = 0.36), and Warrick severity (r = 0.39), as well as with X-ray findings (r = 0.29). Pulmonary function measures also showed significant correlations, with forced vital capacity (FVC) (r = −0.42) and diffusing capacity of carbon monoxide (DLCO) (r = −0.36) (*p* < 0.05).

## 4. Discussion

We conducted a retrospective study on a small series of consecutive SSc patients at a single tertiary academic center to evaluate the usefulness of a portable ultrasound probe in routine screening for pulmonary involvement, specifically SSc-associated interstitial lung disease. B-lines were analyzed in relation to key SSc manifestations, including pulmonary symptoms (dyspnea, cough, Velcro crackles), CT imaging findings (Warrick’s score—total, extent, and severity), and pulmonary function tests. The aim was to validate the role of a handheld US device in the early detection and diagnosis of lung fibrosis associated with SSc.

The management of ILD associated with autoimmune connective tissue diseases, particularly systemic sclerosis, typically follows a standard algorithm involving clinical evaluation, functional testing (spirometry, DLCO), and imaging (X-rays, CT). While high-resolution CT remains the gold standard for screening, diagnosis, and monitoring of SSc-related ILD, as well as for stratifying patients at risk for a progressive fibrosing phenotype, pulmonary ultrasound has also proven valuable for screening and assessing lung parenchyma. Numerous studies have highlighted the usefulness of LUS in scleroderma patients, demonstrating the importance of B-line analysis for detecting SSc-ILD and its correlation with disease severity and extent. The combination of pulmonary function tests and LUS may offer an effective approach for screening ILD as a significant complication in SSc and other autoimmune connective tissue diseases.

At first glance, the profile of SSc patients attending follow-up visits aligns with the literature, particularly middle-aged women with predominantly diffuse forms of the disease. Symptomatic lung involvement was common, with varying degrees of dyspnea reported in up to 74% of cases (more than half classified as NYHA functional class > 2), chronic non-productive cough in one-third, and Velcro crackles detected on pulmonary auscultation in up to half of the patients. Of the seven patients with no abnormalities on chest X-rays, more than half already exhibited subclinical abnormalities on CT scans, indicating ILD. Overall, the majority of cases (n = 16) were classified as SSc with ILD, as confirmed by CT imaging. Positive anti-topoisomerase I antibodies were detected in 14 cases, anticentromere antibodies in three cases, while two cases were seronegative for both ACA and ATA. As a marker of disease activity, low serum complement levels were observed in five cases (26.32%).

We demonstrated mild-to-moderate statistically significant correlations between B-lines on pulmonary ultrasound and clinical manifestations, such as dyspnea and Velcro crackles, showing direct positive correlations (symptomatic patients already had lung involvement detected by imaging). There were also indirect negative correlations with pulmonary function impairment, where an increased number of B-lines was associated with lower pulmonary function, as measured by spirometry and DLCO. Additionally, we observed direct positive correlations between B-lines and corresponding CT imaging findings (*p* < 0.05). As expected, significant differences were found between SSc patients with and without ILD in terms of the number and distribution of B-lines (*p* < 0.05). Furthermore, patients with diffuse cutaneous SSc (dcSSc) and limited cutaneous SSc (lcSSc) exhibited distinct patterns and numbers of B-lines (*p* < 0.05).

Similar correlations have been observed in other studies, often more pronounced than in ours. For example, Cakir Edis et al. (2016) demonstrated statistically significant positive correlations between HRCT findings (as measured by the Warrick score) and the number of B-lines, reinforcing the utility of LUS in the long-term follow-up of SSc-related ILD. This highlights LUS as a valuable tool for monitoring disease progression and managing pulmonary involvement in systemic sclerosis [27].

Furthermore, Gargani et al. (2020) demonstrated in a large cohort of SSc patients across three Italian rheumatology centers that lung ultrasound B-lines are significantly associated with both the development and progression of pulmonary involvement related to scleroderma. As anticipated, the number of B-lines was notably higher in patients with diffuse cutaneous SSc and seropositive anti-topoisomerase I antibody disease, and correlated with lung fibrosis detected on HRCT scans. A cut-off of five B-lines was used to identify patients at risk of developing or worsening ILD. Additionally, >5 B-lines in the posterior thorax, combined with ATA positivity, were linked to new or worsening ILD in patients who already had ILD at baseline [28].

Bruni and colleagues (2022) underscored the role of LUS in detecting interstitial lung disease in systemic sclerosis patients, demonstrating its utility not only for screening pulmonary involvement but also for assessing the extent and severity of the disease. They found that the total number of B-lines was significantly higher in patients with ILD compared to those without, with posterior B-lines being particularly useful for detecting limited lung involvement. In contrast, anterior B-lines were more indicative of distinguishing between extensive and limited ILD. Additionally, a strong correlation between B-lines and CT scores, particularly with the Wells score, was observed. This CT assessment utilized a complex protocol that included qualitative, semi-quantitative, and quantitative evaluations, as well as histogram-based densitometry [29].

More recently, Huang et al. (2023) demonstrated a significant negative correlation between the number of B-lines and total lung capacity, while noting a non-significant relationship with other lung function indices. Additionally, a strong direct correlation was observed between B-lines and both HRCT findings and the Warrick score, suggesting that LUS, in conjunction with HRCT, can provide complementary information for ILD screening and for assessing disease severity in lung fibrosis related to connective tissue diseases [17].

To the best of our knowledge, this study represents the first approach to using lung ultrasound with portable probes, not only in our tertiary scleroderma center but also within our country. While other studies have explored the effectiveness of portable LUS for assessing ILD, they have indicated that LUS can sensitively detect ILD in early systemic sclerosis, even when HRCT scans appear normal [17]. Moreover, once portable ultrasound devices have been validated for accuracy comparable to cart-based machines, recent research has compared commonly available point-of-care handheld devices based on image quality, ease of use, and overall expert satisfaction. The study concluded that, although no single handheld device in the U.S. meets all criteria, the most critical feature remains the quality of the acquired image.

In our study, we explored the utility of a portable, handheld Lumify™ (Philips Ultrasound, Inc, Bothell, WA, USA) probe, which provided excellent image quality and employed a modern approach to B-lines by color-coding their number (green, orange, red). We used a predefined threshold, with “red, 3+” B-lines, to define fibrotic-type interstitial lung involvement. Cut-off values for the number of B-lines used to define ILD have varied across studies, ranging from “>5” (proposed by Picano et al.) to “>10” (proposed by Gargani et al.), and even “>20”, with each threshold showing sensitivity and specificity in correlating B-line numbers with ILD [17].

We did not calculate the sensitivity and specificity of the cut-off value (>3 B-lines) used to define the presence of ILD with our ultraportable Lumify™ (Philips) probe. Instead, we relied on color codes and the number of B-lines to identify fibrotic parenchymal lesions. However, we successfully demonstrated the usefulness of lung ultrasound, using a portable probe at the patient’s bedside, for both screening pulmonary involvement and early detection of fibrosis in asymptomatic patients, as well as for evaluating previously diagnosed fibrotic conditions.

The current study had several limitations, including the small sample size and the heterogeneous patient population, which could have affected the lung imaging results, whether by CT scan or LUS. Additionally, not all lung assessments (PFTs, CT, LUS) were performed simultaneously; however, we believe that a 3-month interval is unlikely to significantly influence ILD progression, even in patients with a progressive fibrosing phenotype. Another potential limitation was that only one LUS-trained rheumatologist conducted the ultrasounds. Lastly, performing a control LUS using a cart-based device in the radiology department would have provided valuable comparative data. Comparing the results of the handheld ultrasound device with a high-performance ultrasound system would indeed enhance the evaluation.

## 5. Conclusions

In conclusion, our study demonstrates the potential value of lung ultrasound as a useful, non-invasive tool in assessing lung involvement in SSc patients. B-line patterns correlated with clinical symptoms, pulmonary function tests, and CT findings, offering a complementary approach to traditional imaging methods like HRCT. Lung ultrasound may play a significant role in identifying and monitoring interstitial lung disease in SSc, particularly due to its accessibility, ease of use, and lack of radiation exposure, providing a valuable addition to the comprehensive evaluation of patients with systemic sclerosis.

## Figures and Tables

**Table 1:** Medsger severity scale for lung involvement.

Organ System	0 (Normal)	1 (Mild)	2 (Moderate)	3 (Sever)	4 (End Stage)
lung	DLCO > 80% FVC > 80% No fibrosis on radiograph SPAP > 30 mhg	DLCO = 70–80% FVC = 70–80% basilar rales fibrosis on radiograph SPAP 35–49 mhg	DLCO = 50–69 FVC = 50–69% SPAP 50–69mhg	DLCO < 50% FVC < 50% SPAP > 65 mhg	oxygen required

DLCO, diffusing capacity of the lungs for carbon monoxide; FVC, Forced vital capacity; SPAP, systolic pulmonary artery pressure.

**Table 2:** Warrick’s semi-quantitative scoring method for HRCT involvement (adapted from [24]).

Severity Score ^a^		Extent Score ^b^	
Abnormality	Grading	Bronchopulmonary Segments—For Each Abnormality, Score by Number of Segments Involved	Grading
Ground-glass opacity	1	1 to 3 segments involved	1
Irregular pleura	2	4 to 9 segments involved	2
Septal/subpleural lines	3	>9 segments involved	3
Honeycombing	4	N.B. extent of disease measured for each of the abnormalities	
Subpleural cysts	5		
Maximal severity score	15	Maximal extent score	15

^a^ Each abnormality detected on HRCT is assigned a point value, with a maximum score of 15 if all abnormalities are present. ^b^ Disease extension is assessed by counting the number of bronchopulmonary segments involved, contributing a total score of up to 15 points. The overall score, ranging from 0 to 30, is calculated by summing the scores for the five basic HRCT abnormalities and the disease extension.

**Table 3:** Distribution and anatomical landmarks for lung ultrasound assessment.

Area		Lines
Upper	R1 L1	Mid-clavicular
Lower	R2 L2	
Upper	R3 L3	Mid-axillar
Lower	R4 L4	
Upper	R5 L5	Paravertebral
Lower	R6 L6	

R, right; L, left.

**Table 4:** Demographic, clinical, and serological profiles of the patients studied.

Variable		Values
Age (years) *		55.26 (7.17)
Gender **	Women	13 (68.42)
	Men	6 (31.58)
Disease duration (years) *	Mean (SD)	6.42 (4.15)
	0–5 (years) **	10 (52.63)
	6–10 (years) **	7 (36.84)
	>10 (years) **	2 (10.53)
Disease subtype **	Diffuse SSc	11 (63.16)
	Limited SSc	8 (36.84)
Raynaud’s phenomenon **		19 (100)
Raynaud condition score	1–5	16 (84.21)
	5–10	3 (15.79)
Modified Rodnan skin score */**	Mean (SD)	10.63 (6.63)
	<10	11 (57.89)
	10–20	6 (31.58)
	>20	2 (10.53)
Digital Pitting Scar **		8 (42.11)
Digital Ulcers (ever) **		4 (21)
Dry chronic cough **		6 (31.58)
Dyspnea **		14 (73.68)
Velcro **		8 (42.11)
EUSTAR score *		2.08 (1.94)
Leukopenia **		7 (36.84)
Anti-SCL-70 antibodies **		14 (73.68)
Anti-centromere antibodies **		3 (15.79)
Complement consumption **		5 (26.32)

* mean (standard deviation, SD); ** n (%) (n = number cases)

**Table 5:** PFT, CT, and LUS assessments in SSc patients.

Parameters	SSc Cohort	SSc with No ILD	SSc with Limited ILD	SSc with Extended ILD	*p*
PFTs (%) (mean +/− SD)					
FVC	100.19 +/− 20.35	103.65 +/− 21.28	97.27 +/− 23.65	88.24 +/− 19.16	<0.05
FEV1%	99.24 +/− 20.07	101.21 +/− 18.12	92.12 +/− 19.77	80.23 +/− 20.21	<0.05
DLCO%	82.51 +/− 19.56	85.47 +/− 18.35	78.55 +/− 15.52	55.43 +/− 12.32	<0.05
LUS					
B-lines (n)	100%				
n region with B-lines	5.42 +/− 2.60	7.45 +/− 1.23	7.54 +/− 3.71	14.4 +/− 5.06	<0.05
Green	6.68 +/− 2.61	7.45 +/− 1.23	5.23 +/− 2.17	7.15 +/− 2.28	<0.05
Orange	5.21 +/− 2.46	none	2.31 +/− 1.54	7.20 +/− 2.34	<0.05
Red	0.10 +/− 0.44	none	none	0.10 +/− 0.44	<0.05
CT					
Warrick’s extension	2.26 +/− 1.91	1.82 +/− 0.78	2.01 +/− 1.23	3.57 +/− 1.34	<0.05
Warrick’s severity	3.31 +/− 2.84	2.00 +/− 1.21	2.23 +/− 1.45	4.23 +/− 1.91	<0.05
Warrick’s total	5.57 +/− 4.47	3.67 +/− 1.42	4.28 +/− 2.03	7.90 +/− 2.01	<0.05

## Data Availability

The data presented in this study are available on request from the corresponding author. The data are not publicly available due to the institution’s confidentiality policy.
